# Erector spinae plane block in laparoscopic nephrectomy as a cause of involuntary hemodynamic instability: A case report

**DOI:** 10.1002/ccr3.4026

**Published:** 2021-03-31

**Authors:** Antonio Coviello, Ludovica Golino, Alfredo Maresca, Maria Vargas, Giuseppe Servillo

**Affiliations:** ^1^ Department of Anesthesiology and Intensive Care Medicine Policlinico ‐ Federico II University Hospital Naples Italy

**Keywords:** erector spinae plane block, erector spinae plane, ESP anesthesia, ESP case report

## Abstract

The Ultrasound‐guided erector spinae plane block (US‐ESPB), used as an anesthesiological block for opioid‐sparing approach and for postoperative analgesia, could represent an involuntary cause of hemodynamic instability. This hemodynamic instability is accentuated by a greater diffusion of local anesthetic in the epidural space.

## Graphical abstract

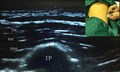

## 1 INTRODUCTION

Multimodal anesthesia that combines the use of an epidural catheter and general anesthesia (GA) is a common technique used for moderate/intense postoperative pain. The technique is considered a quality standard because it provides good control of the anticipated pain. Placement of the epidural catheter is not always possible, however, due to technical difficulties or patient‐related conditions.^1^ A similar result can be achieved through GA combined with locoregional anesthesia techniques, such as the erector spinae plane block (ESPB). ESPB is an interfascial block consisting in an injection of local anesthetic (LA) in a plane between the transverse process and the erector spinae muscles group.^2^ It was originally introduced as analgesia of thoracic neuropathic pain.^3^ Since then, the interest about ESPB has been growing for a range of procedures. Studies show that the efficacy of ESPB is based on the anesthetic spreading partly in the paravertebral space and subsequently—through the intervertebral foramina—in the epidural space, leading to blockage of both somatic and visceral pain.^4^, ^5^, ^6^ ESPB combines some favorable characteristics: simplicity, safety, effectiveness, and spread on several neurotomes.^7^ In the literature, ESPB has also been used for postoperative analgesia in laparoscopic surgery.^8^, ^9^, ^10^ ESPB is ultrasound‐guided, usually, a high‐frequency linear ultrasound (US) transducer is used for the thoracic level and a convex transducer for the lumbar level.^10^, ^11^ The in‐plane or out‐of‐plane needle approach should be used according to the physician's experience, although the in‐plane technique is most frequently used.^11^ Injecting the LA solution should create an anechoic space between the transverse process and the erector spinae muscles; LA should spread out in both caudal and cephalic directions.^12^, ^13^ ESPB can be performed at T4‐T5 level for breast and thoracic surgery and T7‐T8 levels for abdominal surgery.^3^, ^4^, ^5^, ^6^, ^7^ The position chosen for ESPB procedure depends on the type of technique and the application. It is possible that the patient's position may affect the spread of LA.^10^, ^13^ We will evaluate the analgesic efficacy of ESPB in a laparoscopic nephrectomy with the possibility of an opioid‐sparing strategy. In our case, furthermore, we report a significant side effect.

## 2 CASE REPORT

**FIGURE 1:**
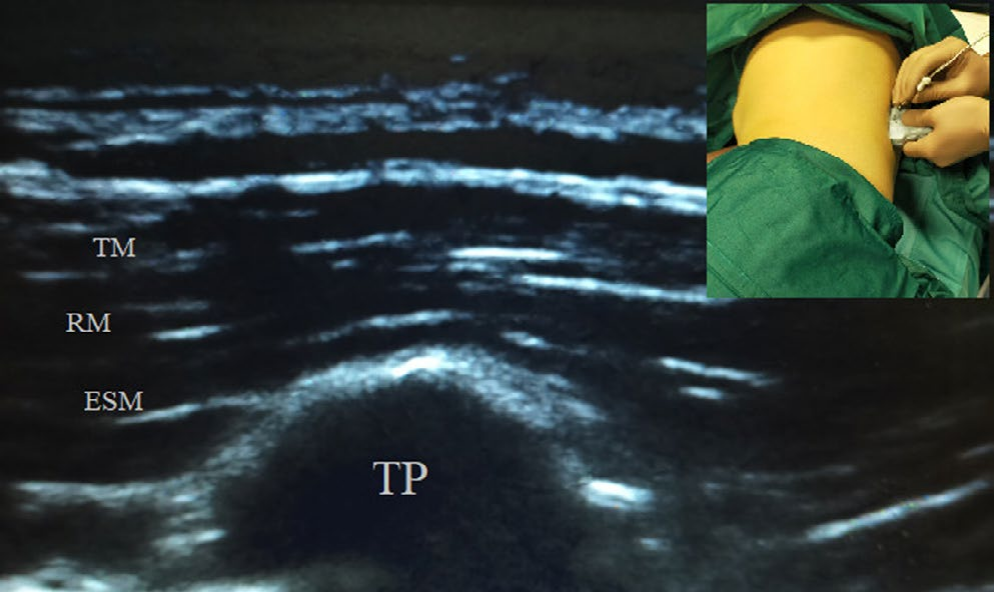
Ultrasound anatomy. ESM, erector spinae muscle; RM, rhomboid major muscle; TM, trapezius muscle; TP, transverse process. At the top left out of plane procedure, lateral to medial approach

We describe the case of about 50‐year‐old male patient with a clear renal cell carcinoma scheduled for laparoscopic right nephrectomy. The patient weighed 70 kg, and his BMI was 24 kg/m^2^. His comorbidities were arterial hypertension; medicated coronary stent positioned 3 years earlier for ischemic heart disease. He reported a good tolerance to moderate physical activity (METS = 5‐7, Class NYHA II). He underwent a preoperative echocardiogram that revealed a normal Left Ventricular Ejection Fraction (55%). An opioid‐sparing approach was selected, suitable for a patient with renal and cardiac comorbidities. We chose combining GA and ESPB. The following parameters were monitored as follows: SpO2, ECG, NIBP, TOF, and BIS. We induced GA with Remifentanil 0.05 μg/kg/min, Propofol 2 mg/kg, and Rocuronium 0.6 mg/kg, and we practiced IOT. Remifentanil was stopped 5 minutes after IOT, and Desflurane 0.9 MAC was used to maintain GA. The initial vital parameters were as follows: SpO2 99%, NIBP 130/80 mm Hg, HR 70 bpm, TOF 0, and BIS 40‐50. The patient was placed on the left side and unilateral right ultrasound‐guided ESPB was performed in this position. We used a high‐frequency linear probe (Sonosite HLF38x 13‐6 MHz, Fujifilm Sonosite Europe). We placed the probe in a transverse orientation to identify the spinous process, then the probe was moved 3 cm laterally until the transverse process was identified. We counted the lamina in the caudal‐to‐cephalad direction, starting from the sacrum and using US to identify the vertebral level, which was then marked with a surgical pen. A 22‐gauge 70 mm Sonoplex Pajunk block needle was inserted out‐of‐plane, with a lateral‐to‐medial direction at T8 transverse process level, until the tip was placed into the plane to deep of the erector spinae muscle (Figure 1). After hydro‐localization with 3 ml of 0.9% saline to open the plane, 20 mL of 0.5% Ropivacaine with Dexamethasone 8 mg were injected. The surgery began and lasted 80 minutes. About 10 minutes after the administration of the ESPB an episode of moderate hypotension occurred (NIBP 80/50 mm Hg, HR 60 bpm), therefore, we reduced Desflurane to a MAC of 0.7 and we increased the rate of fluids administration. After 5 minutes, there was no positive response to the measures taken, indeed the patient showed severe hypotension (50/30 mm Hg) with weak carotid pulse, which led to reflex bradycardia (HR 30 bpm). We administered ephedrine boluses (0.3 mg/kg per bolus, total 25 mg). Stable hemodynamics were restored (NIBP 100/60 mm Hg, HR 75 bpm) and the surgery continued. BIS value was always between 40 and 60. There were no further complications. On awakening, the patient presented RASS 0 and VAS 0, therefore, no analgesics were administered. He did not report nausea, vomiting, shivering, or other side effects. Bromage score, Pinprick test, and Ice test were performed after awakening. These revealed persistence of bilateral sensory block (Hollmen grade III: touch sensation under 30%), extended from T2 to L4; no motor limitation (Bromage grade IV: full flexion of knees and feet). In the first 24 hours the RASS and VAS were both consistently 0, without needing any rescue analgesic, opioids, NSAIDs, neither paracetamol nor nausea nor vomiting occurred; Pinprick tests and Ice tests were repeated every six hours, showing gradual reduction in the area affected by the sensory block. After 12 hours the sensory, block was extended from T6 to L1 and was Hollmen grade II (weak sensation of touch). After 24 hours, there was no residual sensory block (Hollmen grade I: full sensation of touch). The patient was able to stand up after 24 hours.

## 3 DISCUSSION

ESPB is a valid alternative of multimodal anesthesia, both for open and laparoscopic surgery, especially in an opioid‐sparing perspective, as reported by Chin et al, on obese patients.^5^ As this patient was 70‐75 and had cardiac comorbidities, minimizing opioids was deemed advantageous. The studies of Vidal et al and Chin et al on cadavers, and the studies of Swhartzmann et al, on magnetic resonance images showed that volume of 20 ml of fluid performed at T7 transverse process cranially spreads till C7‐T2 vertebra levels and, caudally, till L2‐L3 vertebra levels.^3^, ^4^, ^13^ We, therefore, injected 20 ml of anesthetic solution at T8 level and observed a similar metameric distribution (T2‐L4 on patient's awakening). This wide metameric distribution confirms the spread of the LA in the epidural space, as supported by Forero, Schwartzmann, Vidal, Chin et al^2^, ^3^, ^4^, ^5^ To locate T8, we counted the lamina starting from the sacrum using the ultrasound method illustrated by Selvi in his study.^14^ ESPB gave effective analgesia, and it is simple and safe to perform,^15^ but there may be complications related to the spread of the LA, such as an unexpected motor block described by Selvi et al^16^ Side effects can occur because the amount of LA that will spread in the epidural space is not completely controllable and predictable.^17^ For this reason, in our opinion it would be appropriate to identify any variables favoring LA spread, in order to standardize the ESPB procedure as much as possible, such as patient's position or needle entry mode, as claimed by Tulgar et al, Milone et al and Tsui et al^9^, ^12^, ^18^ In the case we reported, there were severe intraoperative hypotension and bradycardia, peaking at 15 minutes from the execution of ESPB and requiring inotropes. We believe that this side effect is also attributable to a wide spread of LA. Elements supporting this issue are as follows: the onset time of hypotension (15 minutes after performing ESPB), compatible with the onset of the block, the absence of pain both when the patient woke up and throughout the first day, and the persistence of sensory block (grade III Hollmen on awakening) for 18 hours after waking up. The sensory block was bilateral, although we performed ESPB only on the right side, and this is a further indication of the epidural diffusion of the LA. We, therefore, think that some factors contributed to this important LA spread:

1. performing block in lateral position;
2. intra‐abdominal pressure given by pneumoperitoneum, immediately after the execution of ESPB (5 minutes), which was, in our opinion, the determining casual factor;
3. out‐of‐plane ultrasound approach, with lateral‐to‐medial needle entry.

## 4 CONCLUSIONS

Intraoperative hypotension and bradycardia can be dangerous, particularly in older patients with cardiac comorbidities. For this reason, although clinical investigations are needed regarding what we reported, we consider useful to standardize the ESPB technique to reduce complications. Our idea, based on our experience and on literature, is that the cranial‐caudal needle approach could reduce the occurrence of these adverse effects and improve the metameric spread of LA. We, therefore, believe that ESPB should be performed at least 20 minutes before patients are placed in a surgical position, especially if the patient undergoes laparoscopic surgery. The increase in abdominal pressures caused by pneumoperitoneum was probably the most important factor favoring the spread of LA.

## CONFLICT OF INTEREST

None declared.

## AUTHOR CONTRIBUTIONS

All persons who meet authorship criteria are listed as authors, and all authors certify that they have participated sufficiently in the work to take public responsibility for the content, including participation in the concept, design, analysis, writing, or revision of the manuscript. Furthermore, each author certifies that this material or similar material has not been and will not be submitted to or published in any other publication before its appearance in the Clinical Case Reports. AC and LG: involved in conceptualized and designed the study. AC, LG, and AM: acquired the data. MV and GS: analyzed and/or interpreted the data. AC, LG, MV, and AM: drafted the manuscript. AC and GS: revised the manuscript critically for important intellectual content. AC, LG, AM, MV, GS: approved the version of manuscript to be published.

## CONSENT FOR PUBLICATION

Written informed consent was obtained from the patient for publication of this case report and its accompanying image. A copy of the written consent is available for review by the Editor‐in‐Chief of this journal.

## Data Availability

The authors confirm that the data supporting the findings of this study are available within the article and its supplementary materials.
